# Reports of a Series of Inoculations for the Variolæ Vaccinæ, or Cow-Pox; with Remarks and Observations on the Disease, Considered as a Substitute for the Small-Pox

**Published:** 1799-06

**Authors:** William Woodville

**Affiliations:** Physician to the Small-Pox and Inoculation Hospitals


*Reports of a Series of Inoculations for the Variolæ Vaccinæ, or Cow-Pox; with Remarks and Obfervations on the Difeafe, confidered as a Subftitute for the Small-Pox:*— By WILLIAM WOODVILLE, M.D. Phyfician to the Small-Pox and Inoculation Hofpitals. 8vo. 156. p. *London.* Phillips and Son.

DR. WOODVILLE introduces his work with an account of Dr. JENNER's firft publication, and the impreffion it made upon him. In confequence of experiments made with the matter of greafe at the Veterinary College, Mr. SIMMONS's Experiments, and Dr. PEARSON's Inquiry, p. 83, 84, &c. Dr. Woodville concludes, that the vaccine difeafe is not derived from the horfe.

"But though Dr. Jenner feems to have been mifled with refpect to the origin of the cow-pox, ftill his facts and obfervations concerning its effects upon mankind, are not lefs valid and important; nor did I feel the lefs defirous to try how far they would be invalidated or confirmed by a more enlarged experience than he had the opportunity of acquiring.

At p. 12 is given an account of the cafe of Sarah Rice, a milker, of which a reprefentation is attempted in the plate defigned for our firft Number.

Dr.

Dr. Woodville then proceeds, " Before relating the cafes of inoculation with the matter of cow-pox, I have judged it proper in the firft place briefly to ftate what are the local effects produced by inoculating variolous matter, fo that the progrefs of the infection in both cafes may be compared, and the fubject of inoculation at large, be better underftood.

" In cafes wherein inoculation of the fmall-pox proves effectual, a fmall particle of variolous matter being applied by a fuperficial puncture of the fkin, ufually produces in the courfe of three or four days, or fooner, a little elevation of the punctured part, difcoverable by the touch, and a red fpeck diftinguifhable by the eye. From this time the rednefs advances in a circular form, more or lefs rapidly, according to the conftitutional circumftances of the patient; and the firft effect of this fuperficial inflammation is the formation of a vefìcle upon its centre, which ufually appears between the fourth and feventh day after the inoculation. The extent of this vefìcle is generally found to bear fome proportion to the intenfity of the inflammation; and contains a limpid fluid, by the abforption of which the fmall-pox is produced. The vefìcle foon burfts, and the central part of the puncture becomes depreffed, and often of a dark hue; which appearances, together with the marginal inflammation, continue to increafe, till the eruptive fymptoms fubfide, when the edges of the depreffed part begin to fwell with a purulent fluid, and the inflammation gradually recedes.

" Thus it appears that the variolous matter, firft inferted by the puncture, like that of other morbid poifons, is not capable of being immediately abforbed, but lodges in the fkin, and there excites an inflammatory procefs, by which a new matter, producing the difeafe, is generated*. It would feem alfo, that this procefs is carried to a greater or lefs extent in different perfons before the matter enters the abforbents, owing probably to the greater or lefs aptitude in thefe veffels to receive it. Hence we find the local inflammation in fome cafes confiderably advanced, before the fyftem becomes affected; while in others the eruptive fymptoms fupervene, when it appears to have made but very little progrefs; and therefore, though the eighth day after the inoculation proves the ufual period at which the patient feels indifpofed, yet this frequently happens much fooner or later, and the progrefs of the cow-pox infection will be found to take the fame latitude.

" Monday, January 21, 1799, I took the matter of cow-pox, in a purulent ftate, from the teats of a cow, with which I immediately inoculated feven

* " In the second Volume of the 'History of Inoculation,' (now nearly ready for the press) I have endeavoured to shew that the general greater mildness of the inoculated than the casual small-pox, depends upon this circumstance."

feven perfons by a fingle puncture in the arm of each, or rather by fcratching the fkin with the point of the lancet, till the inftrument became tinged with blood."

Dr. Woodville then commences his detail of the cafes which he inoculated between the 21ft of January, 1799, and the 18th of March following, amounting to *two hundred.*

Nearly the whole of thefe perfons were fubfequently inoculated with variolous matter, and many of them expofed to perfons labouring under this difeafe, without a fingle inftance of the fmall-pox being produced, after the vaccine infection had taken effect. The *forty-ninth* cafe is that of Mr. Walker's child, given in our fecond Number.

For the ufual courfe of the cow-pox we refer our readers to the work itfelf, and the account we have given in our preceding Numbers.—At p. 115 Dr. Woodville gives a tabular ftatement of the progreffive defcent of the cow-pox infection from patient to patient, as well as the magnitude of the difeafe which was excited by the inoculation. From the table, which extends much beyond the detail of cafes, we collect the following *Facts:*

1. That out of about 500 cafes of the inoculated cow-pox, one proved fatal; and that in fome others the difeafe, from the number of puftules, was of formidable feverity; while, on the other hand, a very large proportion of the patients were fcarcely difordered from the inoculation, and had no puftules. p. 149.

2. That the matter of the vaccine difeafe has generally produced much fewer puftules and lefs indifpofition than that of the fmall-pox. p. 150.

3. That about two-fifths of all the perfons inoculated for the variolæ vaccinæ had no puftules; and that in not more than a fourth part of them was there experienced any perceptible diforder of the conftitution. *Ibid.*

"But it muft be acknowledged," continues Dr. Woodville, "that in feveral inftances the cow-pox has proved a very fevere difeafe. In three or four cafes, out of 500, the patient has been in confiderable danger, and one child, as I have already obferved, actually died under the effects of the difeafe. Now, if it be admitted, that at an average, one of 500 will die of the inoculated cow-pox, I confefs I fhould not be difpofed to introduce this difeafe into the Inoculation Hofpital, becaufe out of the laft 5000 cafes of variolous inoculation, the number of deaths has not exceeded the proportion of one in 600. But I am inclined to think, that if the matter of the cow-pox ufed for the purpofe of inoculation were only taken from thofe in whom the difeafe appeared in a very mild form, the refult would be more favourable favourable than in the ſtatement here given. For though it has occaſionally happened that the matter taken from the arm of a patient, in whom the diſorder neither produced fever nor eruption, has in others produced both; yet ſtill it has much more commonly had the effect of exciting a milder diſeaſe than the matter of the puſtules, or than that which was obtained from a patient who had the diſeaſe in a ſevere manner, as may be ſeen by an examination of the table.

